# *In vitro* investigations of red blood cell phase separation in a complex microchannel network

**DOI:** 10.1063/1.5127840

**Published:** 2020-01-02

**Authors:** A. Mantegazza, F. Clavica, D. Obrist

**Affiliations:** 1 ARTORG Center for Biomedical Engineering Research, University of Bern, 3010 Bern, Switzerland; 2 Integrated Actuators Laboratory, École Polytechnique Fédérale de Lausanne (EPFL), 2002 Neuchâtel, Switzerland

## Abstract

Microvascular networks feature a complex topology with multiple bifurcating vessels. Nonuniform partitioning (*phase separation*) of red blood cells (RBCs) occurs at diverging bifurcations, leading to a heterogeneous RBC distribution that ultimately affects the oxygen delivery to living tissues. Our understanding of the mechanisms governing RBC heterogeneity is still limited, especially in large networks where the RBC dynamics can be nonintuitive. In this study, our quantitative data for phase separation were obtained in a complex *in vitro* network with symmetric bifurcations and 176 microchannels. Our experiments showed that the hematocrit is heterogeneously distributed and confirmed the classical result that the branch with a higher blood fraction received an even higher RBC fraction (*classical partitioning*). An inversion of this classical phase separation (*reverse partitioning*) was observed in the case of a skewed hematocrit profile in the parent vessels of bifurcations. In agreement with a recent computational study [P. Balogh and P. Bagchi, Phys. Fluids **30**,051902 (2018)], a correlation between the RBC reverse partitioning and the skewness of the hematocrit profile due to sequential converging and diverging bifurcations was reported. A flow threshold below which no RBCs enter a branch was identified. These results highlight the importance of considering the RBC flow history and the local RBC distribution to correctly describe the RBC phase separation in complex networks.

## I. INTRODUCTION

Living tissues rely on the supply of oxygen and nutrients as well as on the removal of carbon dioxide and other metabolites^21^ to maintain a physiological state. The microcirculation is the system where these mass exchange phenomena are happening. Microvascular networks feature a complex topology with highly interconnected capillaries bifurcating in an irregular fashion.^10,22^ Blood flowing in the microcirculation cannot be treated as a homogeneous Newtonian fluid because it contains deformable red blood cells (RBCs), whose dimensions are comparable to the vessel diameter.^50^ The RBC concentration is heterogeneous in space and time, and there are even RBC-free vessels.^47^ Therefore, the particulate nature of blood and phase separation effects must be considered for a correct interpretation of fluid dynamics at the microscale. In such microvascular networks, the dynamics of RBCs flowing through sequentially diverging and converging bifurcations plays an important role in the local perfusion.

Nonuniform RBC partitioning at diverging bifurcations leads to the heterogeneous RBC distribution in the daughter branches and ultimately impacts the oxygen delivery to living tissues.^41^ The daughter branch with a higher flow rate tends to receive a disproportionally higher RBC fraction (the Zweifach-Fung effect^17^) at the cost of a RBC reduction in the branch with a lower flow rate. The extreme case is a daughter vessel with a very high flow rate drawing all RBCs and leaving the low-flow branch with only plasma. Fung^17^ reported that the RBC flow at microvascular bifurcations is self-regulated: RBCs entering the high-flow branch cause an increased RBC density in this branch, which leads to a higher local resistance. Eventually, this will reduce the flow in this branch to the point when RBCs start entering the other branch. This self-regulating mechanism leads to a reduction of flow disparities between daughter vessels. Schmid *et al.*^49^ showed that such well-balanced bifurcations dominate cerebral microvascular networks in mice and support robust perfusion characteristics. The Zweifach-Fung effect is strongest in capillaries, where the vessel diameter is comparable to the RBC diameter (strong confinement).^50^ In larger vessels, RBCs tend to follow the streamlines of the plasma flow.^6^

Given the high importance of the microcirculation in human physiology, blood flow distribution at microvascular bifurcations has been thoroughly studied over the past decades. Phase separation has been investigated with two-dimensional^6,7,12,55^ and three-dimensional^25^ numerical simulations in symmetric and asymmetric bifurcations. In particular, Barber *et al.*^6,7^ and Doyeux *et al.*^12^ described mechanisms related to the cell partitioning causing the individual cell trajectories to divert from fluid streamlines. *In vitro* experiments have also been reported where the flow partitioning of rigid particles,^46^ lipid vesicles,^12^ or red cells^9,10,14,47,53^ was studied in models of symmetric and asymmetric bifurcations (T-shaped or Y-shaped). Fenton *et al.*^14^ and Roman *et al.*^47^ studied the effects of the feeding hematocrit and the local bifurcation geometry. A reduction of the phase separation for increasing hematocrit was observed in symmetric bifurcations.^47^ Carr and Wickam,^9^ Roberts and Olbricht,^46^ and Sherwood *et al.*^53^ showed how the proximity of two diverging bifurcations increases the asymmetry of the phase separation. Sherwood *et al.*^53^ demonstrated that in the case of weak confinement (particle to channel diameter ratio equal to 0.16), the phase separation asymmetry was due to a skewed hematocrit distribution in the parent vessel of the bifurcations. In an experimental study of RBC partitioning with an asymmetric bifurcation, Clavica *et al.*^10^ observed a reduction and even an inversion of the Zweifach-Fung effect with increasing flow rate.

A number of computational studies have investigated large-scale networks, where the geometry of the networks was either idealized^1,39,48^ or obtained from the reconstruction of *in vivo* images.^5,18,20,31,42,44^ Numerical results of idealized^39^ or realistic^5^ networks showed that the RBC distribution was heterogeneous, and this was even the case if the networks were symmetric and the boundary conditions at the inlet were kept constant.^39^ Balogh and Bagchi^5^ developed a direct numerical simulation technique for blood flow modeling in microvascular networks with *in vivo*-like features. They showed how RBC partitioning could oscillate between classical partitioning (the Zweifach-Fung effect) and reverse partitioning (inversion of the Zweifach-Fung effect^10,52^) over time. Different cellular-scale mechanisms were identified that could affect the local RBC distribution: (I) skewness of the hematocrit profile in the parent branch; (II) RBC lingering at branching points;^4^ (III) local self-regulation due to cell-cell and cell-vasculature interactions; and (IV) flow balancing due to transient capillary obstructions.^5^

In contrast to computational models, *in vitro* studies to investigate the local RBC partitioning for complex and anatomically plausible microvascular networks^15,28,34,54^ are scarce. In particular, there is a lack of experimental research with channel sizes comparable to the RBC diameter (≤10μm), which can probably be attributed to technical challenges of microfabrication.

In the present study, a complex yet idealized capillary network was designed and fabricated. The network topology was inspired by studies previously reported in the literature.^34,35,44,48^ All microchannels had a width of 10μm (particle to channel diameter ratio ∼0.6) to reproduce the typical confinement that RBCs experience in microvascular capillary networks. This allowed us to provide for the first time quantitative *in vitro* data on phase separation in a complex network with many sequential diverging and converging bifurcations for different inflow conditions. This also includes data for bifurcations with RBC-free daughter vessels and information on the flow threshold for RBC-free vessels. It will be shown that the observed phase separation is rather well described by the model of Pries *et al.*,^41^ although some bifurcations exhibited an inversion of the Zweifach-Fung effect. Further, we investigated the relation between the hematocrit distribution in the parent vessels of a series of diverging bifurcations and the RBC partitioning, with a particular interest on the consequences for the Zweifach-Fung effect.

## II. MATERIALS AND METHODS

### A. Microdevice fabrication

We used a microfluidic device comprising a complex honeycomb network of microchannels whose dimensions are inspired by *in vivo* length scales.^33^ A similar design was used by Schmid *et al.*^48^ to perform *in silico* studies and by Merlo^34,35^ for *in vitro* experiments. Microchannels had rectangular cross sections (width=10μm, height=8μm), and a length of 85μm. Wider drainage microchannels (width=100μm, height=8μm) were placed upstream and downstream of the capillary network at the inlet and outlet. The typical range of RBC velocities in the microcirculation (0−2mm/s)^23,36^ was reproduced by a constant pressure head at the inlet of the device.

**FIG. 1.**
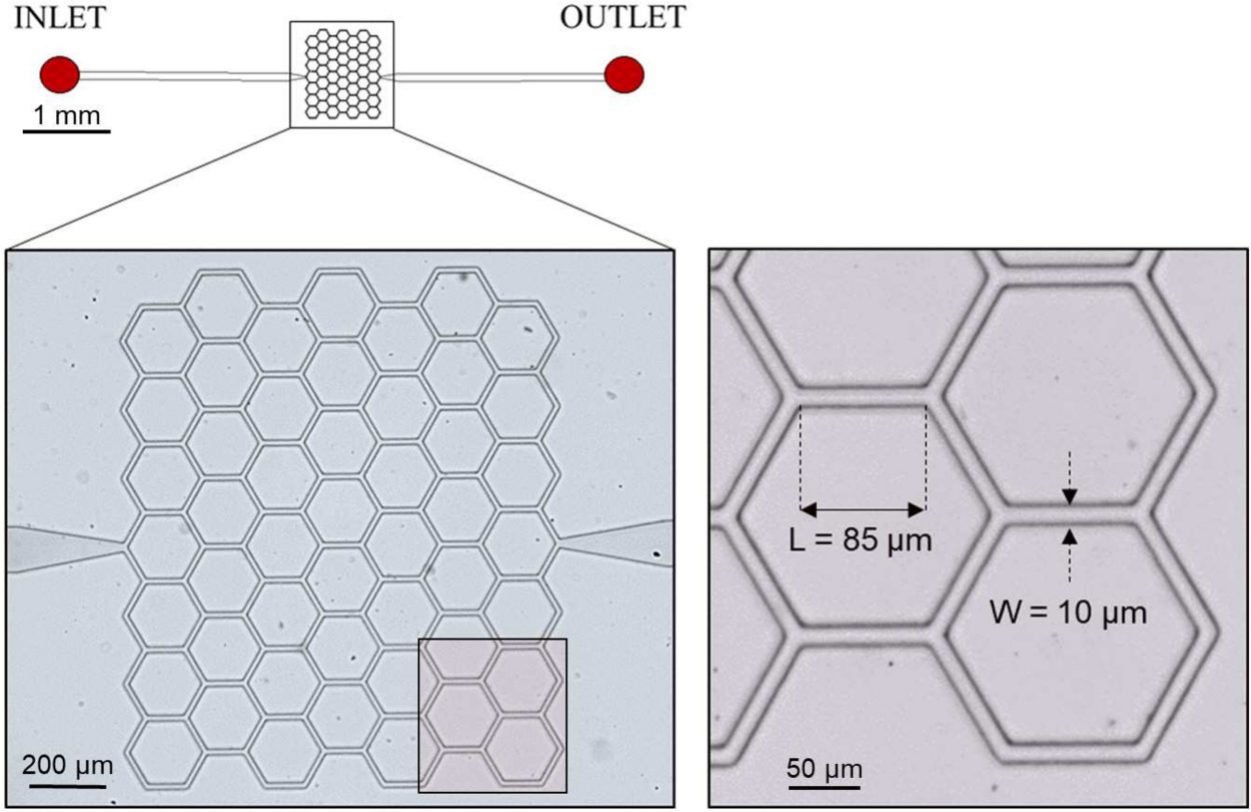
Microfluidic device. Schematic of the microfluidic device (top) and magnified microscope images (bottom). The device had a single inlet and a single outlet and embedded a network composed of 49 hexagonal elements. The general direction of flow was from left to right. All the microchannels had a width W=10μm, a height H=8μm, and a length L=85μm. The image at the bottom right represents a typical microscope field of view of 512×512pixels.

The geometry of the microdevice was designed using DraftSight (Dassault Systèmes, Vélizy-Villacoublay, France) and transferred onto a chrome photomask (JD Photodata, Hitchin, UK). The master of the microdevice was made from a silicon wafer (Prolog Semicor Ltd, Kiev, Ukraine) and fabricated using conventional soft-lithography. The process involved the following steps: coating of the wafer with a SU-8 negative photoresist (thickness=8μm, GM1060 from Gersteltec Sarl., Pully, Switzerland) and patterning by exposure to UV light (Suss MicroTec Lithography GmbH, Garching, Germany) through the photomask. The microfluidic device was then made of polydimethylsiloxane (PDMS) via replica molding by pouring liquid PDMS (Sylgard 184, Dow Corning, Midland, MI, USA) with a monomer/curing agent weight ratio of 10:1 onto the master device. After degassing for approximately 25 min, the microfluidic chip was cured overnight at $60{\;}^{{}^{\circ}}C$. In the final fabrication step, the casted PDMS was bonded to a flat PDMS layer by oxygen-activated plasma treatment (Harrick Plasma, Ithaca, NY, USA). The resulting device had a honeycomblike geometry with 176 microchannels and the actual dimensions (Fig. 1) were measured *a posteriori* using a profilometer and an Axioplan microscope (Carl Zeiss AG, Jena, Germany).

### B. Red blood cell suspension

Fresh venous blood (10 ml) from specific pathogen-free Large White pigs was provided by the Institute of Virology and Immunology (IVI) in Mittelhäusern, Switzerland. The 10 ml blood samples were obtained exclusively in the context of 300–400 ml blood drawings from adult pigs performed for the purpose of *in vitro* studies at the IVI under the animal licenses BE 88/14 and BE131/17 delivered by the veterinary authorities of the Canton of Bern. The samples were centrifuged at 1800×g for 10min to separate cellular components and plasma. The supernatant was discarded, and 0.5 ml of RBCs was collected. A solution of Bovine Serum Albumin (BSA) (Sigma-Aldrich, St Louis, MO, USA) dissolved at 1% in Phosphate-Buffered Saline (PBS) (Sigma-Aldrich, St Louis, MO, USA) was used to wash RBCs to reduce echinocytosis.^45^ After washing, the RBC samples were centrifuged for 5 min at 2500×g.

The suspending medium (hereafter referred to as *plasma*) was prepared to match the density of the RBCs to avoid sedimentation. As suggested by Roman *et al.*,^47^ it contained 65% Glucose-Albumin-Sodium-Phosphate (GASP) buffer (PBS with 5.5 mM glucose and 4% BSA) with 35% of stock solution (90% Optiprep (Sigma-Aldrich, St Louis, MO, USA) + 10% GASP 10 times concentrated), resulting in a density of $1090\;\mathrm{kg}/m^{3}$ and a viscosity of $1.96\times 10^{-3}\;\mathrm{Pa}\;s$ at $20{\;}^{{}^{\circ}}C$, which is in the physiological range of plasma.^17^ RBCs were diluted in the medium such that the hematocrit was 10%. We chose a reservoir hematocrit $H_{\;f}\;=\;10\text{\%}$, which is within the physiological range for microcirculation.^24,40^ Due to the centrifugation stages, no other biological components but RBCs were expected to be in the final suspension. Experiments were carried out at ambient temperature ($T\;=\;21{\;}^{{}^{\circ}}C$) within 12 h after the blood collection to ensure a healthy state of the RBCs for the whole duration of the experiment.

### C. Experimental protocol

**FIG. 2.**
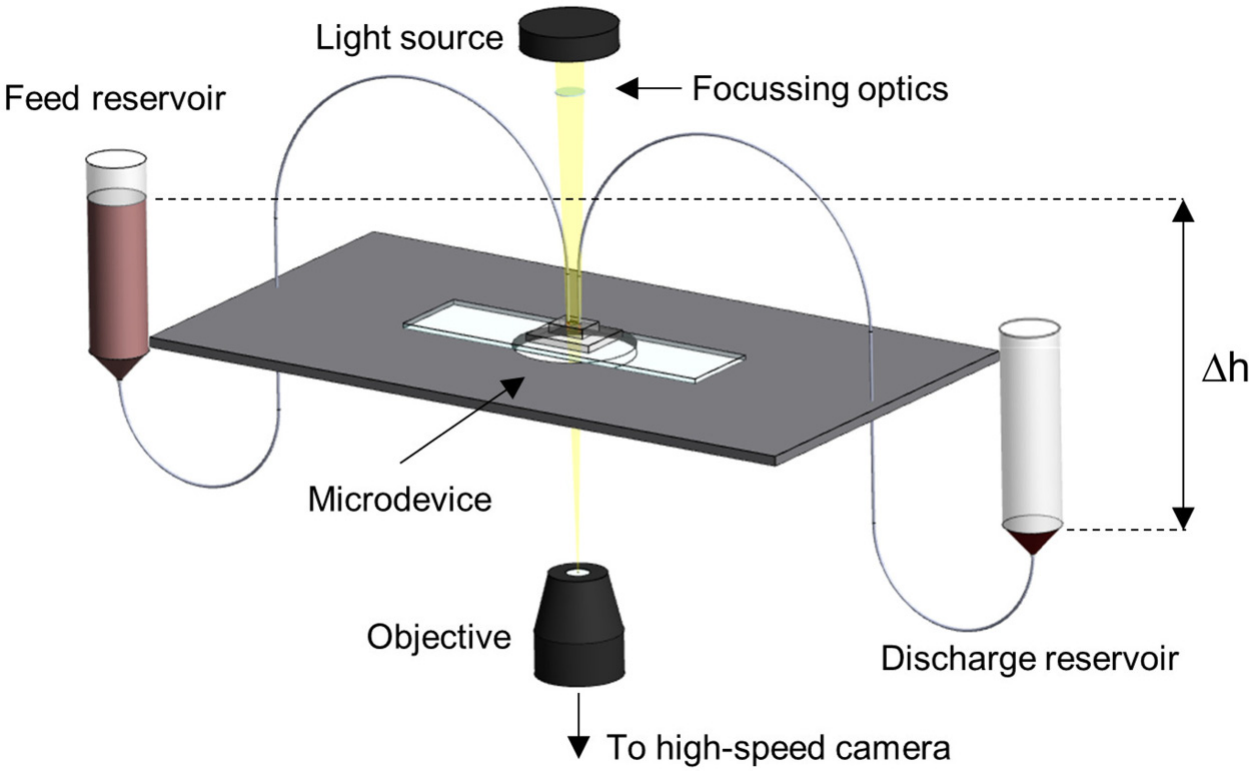
Schematic of the experimental setup. A reservoir containing 10 ml RBC suspension was connected to the inlet of the microchannel. The outlet was at atmospheric pressure. The flow was driven by the hydrostatic pressure difference between the fluid level in the reservoir and the outlet. The hydrostatic pressure difference was controlled by setting the reservoir height via a linear-motion stage (ΔP=ρ×g×Δh).

The microchannel network was degassed and prefilled as described by Clavica *et al.*^10^ The blood sample was placed in a 10 ml centrifuge tube, was mounted on a vertical linear-motion stage, and connected to the inlet of the microdevice. We performed experiments imposing a hydrostatic pressure difference between the fluid level in the reservoir and the device outlet (Fig. 2). Two different driving pressures were used: $\Delta P_{1}\;=\;35.3\;\mathrm{mbar}$ and $\Delta P_{2}\;=\;47.1\;\mathrm{mbar}$ (hereafter referred to as *low velocity* and *high velocity* experiments, respectively). Pressure-driven flows have short response times when the pressure is changed and they are stable over long periods, making the steady-state study of phase separation possible.^47^ Attention was paid to maintain the pressure difference constant throughout the experiments such that pressure variations due to changing fluid levels in the reservoir were negligible. The microdevice was placed on an inverted microscope (Nikon, Japan) with a 10× air objective (NumericalAperture=0.45) and illuminated by a LED lamp. A XYZ micrometer stage was used to focus the light on a region of interest (ROI) of 512×512pixels (Fig. 1). For each experiment, the hydrostatic pressure difference was set first. Once a steady-state condition was reached, videos of 25 s were recorded at 120 frames/s using a high-speed camera (ORCA-flash 4.0, Hamamatsu, Japan).

### D. Image analysis and metrics

**FIG. 3.**
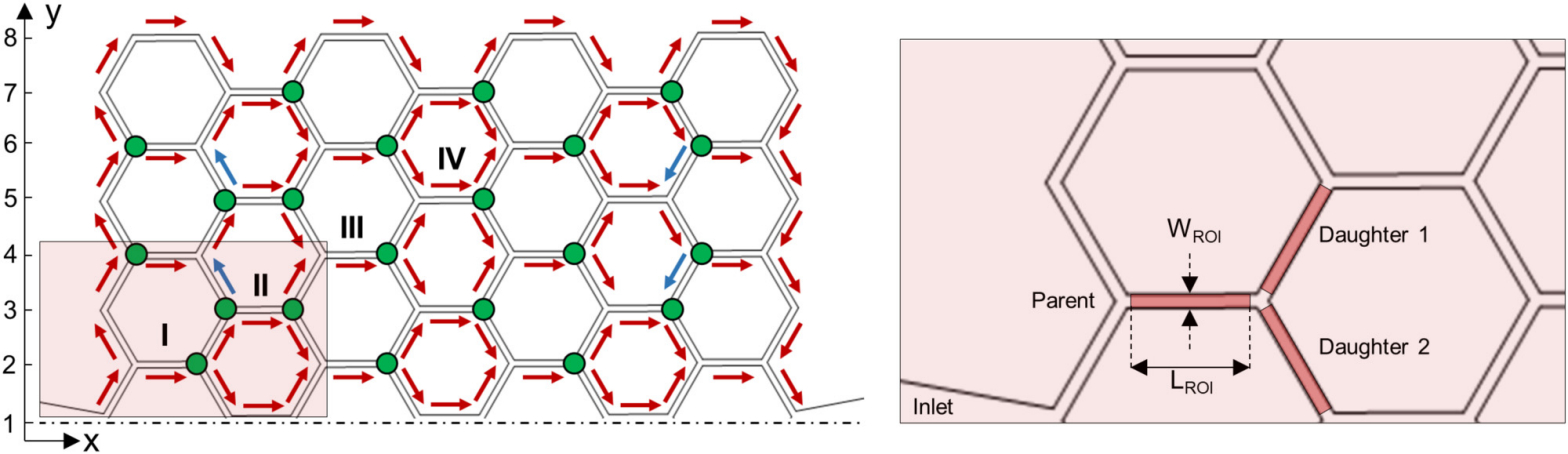
Microfluidic network. Upper half of the microfluidic device (left). Red arrows indicate the direction of blood flow. Blue arrows indicate the direction of plasma flow in RBC-free vessels. Green dots represent diverging bifurcations. The path along I, II, III, and IV features a series of diverging and converging bifurcations, studied in Sec. III C 2. The inset (right) shows an example of a diverging bifurcation. Region of interests (ROIs) for the parent vessel and for the daughter branches are marked in red. The width of the ROI ($W_{ROI}$) is 10μm, while the length ($L_{ROI}$) is 78μm for all vessels of the bifurcation.

The video sequences were imported and preprocessed in Matlab (Mathworks, Natick, MA, USA) to correct for background illumination differences, remove the background, and convert the color from gray scale to black and white. The processed frames were imported in an open-source program for Particle-Tracking Velocimetry (PTV)^8^ (PTVlab, Mathworks, Natick, MA, USA). The output data from PTVlab were postprocessed with Matlab to automatically derive the following quantities for each frame: average RBC velocity [mm/s], RBC line density [RBCs/m] and tube hematocrit ($H_{t}$, i.e., the ratio of the total RBC volume to channel volume). The average RBC velocity is the mean of the velocities of all RBCs identified in the region of interest (ROI, Fig. 3) for a given frame. The RBC line density is the number of red cells present in the ROI for a given frame divided by the length of the channel segment included in the ROI. The raw data for the number of RBCs within the ROI were first smoothed with a second-order Savitzky-Golay filter in Matlab (Mathworks, Natick, MA, USA), using a window of 199 points corresponding to intervals of 1.66 s. These data were then used to compute RBC flux and blood flow rate (see Sec. II D 2). The PTV algorithm was already compared in a previous study^10^ with the line scan method (LSM)^27^ to ensure accurate results. In the present study, we performed a more detailed validation (see Sec. III A). To this end, line scans were sampled from the microchannel and stacked in a temporal sequence. The RBCs were identified from line traces in these stacks and their velocity was computed from the slope of these line traces. Additionally, the data from the particle-tracking algorithm were checked by verifying the mass balance for the RBC flux at bifurcations.

#### 1. Hematocrit measurements

The technique for the measurement of the tube hematocrit $H_{t}$ relied on the identification of the RBCs by PTVlab. The discharged hematocrit in the reservoir was $H_{d}\;=\;10\text{\%}$ and, therefore, the tube hematocrit $H_{t}$ was 10% or less because of the Fåhraeus effect.^13^ This low hematocrit allowed counting of individual RBCs.^47^ The tube hematocrit for each frame could then be calculated as

$$H_{t}\;=\;\frac{N_{rbc}\times MCV_{rbc}}{V_{channel}},$$ (1)

where $N_{rbc}$ is the number of RBCs in the ROI, $V_{channel}$ is the volume of the microchannel segment in the ROI ($V_{channel}\;=\;L_{ROI}\times W_{ROI}\times H$), and $MCV_{rbc}$ is the RBC mean corpuscular volume. The average volume of a single porcine RBC^2,56^ is $MCV_{rbc}\;=\;56\;\mu m^{3}$. The mean hematocrit was computed as the temporal average of the tube hematocrit.

Data on the number and position of RBCs were used to compute line density profiles in the parent vessels of four diverging bifurcations (see Sec. III C 2) in the function of the lateral coordinate (y-coordinate). To this end, the ROI was divided parallel to the axial direction into 18 subregions (each 1 pixel wide). We assigned each RBC to one of those regions depending on its centroid position determined by particle tracking. This allowed us to count the number of RBCs at a given lateral position in the microchannel. Line density profiles were computed by dividing the mean RBC number at each lateral position by the length of the ROI [Fig. 9(b)], where the lateral coordinate was normalized by the width of the ROI ($y*\;=\;y/W_{ROI}$). To quantify the skewness of the line density profiles, the hematocrit skewness index^53^ $S_{h}$ for all divergent bifurcations considered in the study was computed as

$$S_{h}\;=\;\left|\frac{\int_{0}^{0.5}Ht(y*)\;dy*}{\int_{0}^{1}Ht(y*)\;dy*}-0.5\right|,$$ (2)

where $S_{h}\;=\;0$ for a perfectly symmetric hematocrit profile along the width of the microchannel, while $S_{h}\;=\;0.5$ for a completely skewed hematocrit profile.

For further validation, the tube hematocrit measurements obtained from particle tracking were compared to measurements obtained from image sequences, which were postprocessed according to the photometric method by Roman et *al.*^47^ More information on this validation can be found in Appendix B.

#### 2. Determination of RBC flux and blood flow rate

Images of RBCs flowing in the microfluidic network were used to calculate the velocity in the parent and in the daughter branches of the diverging bifurcations. The PTV algorithm allowed to compute the velocity $U_{rbc}(x,y)$ in the plane XY for all the RBCs identified in the region of interest. The mean RBC velocity ($U_{rbc}$) is the mean of the velocities of all RBCs identified in the ROI for a given frame averaged over all frames included in the image sequence. Assuming a mean velocity $U_{rbc}$ in the ROI ($width\;=\;W_{ROI}$, $length\;=\;L_{ROI}$, height=H), we compute the mean RBC flux as

$$Q_{rbc}\;=\;U_{rbc}\times H_{t}\times W_{ROI}\times H\;=\;U_{rbc}\times \frac{N_{rbc}\times MCV_{rbc}}{L_{ROI}}.$$ (3)

Starting from the mean RBC velocity $U_{rbc}$, the total blood flow rate could be calculated according to

$$Q_{blood}\;=\;\chi \times U_{rbc}\times W_{ROI}\times H,$$ (4)

where χ is a coefficient taking into account the velocity difference between the plasma and RBCs. The plasma velocity is typically smaller than the RBC velocity due to the Fåhraeus effect.^13^ However, we assumed χ=1 (similar to Sherwood *et al.*^53^) implying that the mean velocity of the total blood is equal to the mean RBC velocity. We will see below that one of the main results of this study (phase separation diagram, Fig. 8) is independent of the choice of χ.

The RBC flux and blood flow rate data were corrected according to the procedure introduced by Pries *et al.*^41^ and used in other experimental studies.^47,53^ This approach allowed to ensure mass conservation at each bifurcation, to reduce scatter in the experimental data and the uncertainty in the fitting.^41,53^ The corrected flow values (marked by hats) for the generic uncorrected flow rates $Q^{P}$, $Q^{1}$, and $Q^{2}$ were calculated as

$${\hat{Q}}^{P}\;=\;Q^{P}\times \frac{1+W_{1}+W_{2}}{W_{P}+W_{1}+W_{2}},$$ (5)

$${\hat{Q}}^{1}\;=\;Q^{1}\times \frac{W_{P}+(Q^{P}-Q^{2})/(Q^{P}+Q^{2})+W_{2}}{W_{P}+W_{1}+W_{2}},$$ (6)

$${\hat{Q}}^{2}\;=\;Q^{2}\times \frac{W_{P}+(Q^{P}-Q^{1})/(Q^{P}+Q^{1})+W_{1}}{W_{P}+W_{1}+W_{2}},$$ (7)

where the weighting factors were given by

$$W_{P}\;=\;\frac{Q^{P}}{Q^{1}+Q^{2}},$$ (8)

$$W_{1}\;=\;\frac{Q^{1}}{Q^{P}+Q^{2}},$$ (9)

$$W_{2}\;=\;\frac{Q^{2}}{Q^{P}+Q^{1}}.$$ (10)

The fractional RBC flux ($\Psi^{i}$) and the fractional blood flow ($\Phi^{i}$) in the daughter branch i are given by

$$\Psi^{i}\;=\;\frac{{\hat{Q}}_{rbc}^{i}}{{\hat{Q}}_{rbc}^{P}},$$ (11)

$$\Phi^{i}\;=\;\frac{{\hat{Q}}_{blood}^{i}}{{\hat{Q}}_{blood}^{P}},$$ (12)

where i=1,2 denotes the daughter branch 1 and 2 of a generic diverging bifurcation (Fig. 3). Note that the coefficient χ defined in Eq. (4) cancels in the definition of $\Phi^{i}$, such that the phase separation results are independent of the choice of χ.

The measured values for $\Psi^{i}$ and $\Phi^{i}$ were compared with the phase separation law proposed by Pries *et al.*,^42^ which is defined as

$$\Psi_{Pries}^{i}\;=\;\left\{ \begin{array}{cc} 0\; & \mathrm{if}\;\Phi^{i}<X_{0},\;\\ 1\; & \mathrm{if}\;\Phi^{i}>1-X_{0},\;\\ \frac{1}{1+\exp\left(-\left(A+B\;logit\left(\frac{\Phi^{i}-X_{0}}{1-2X_{0}}\right)\right)\right)}\; & \mathrm{else},\; \end{array}\right.$$ (13)

where A, B, and $X_{0}$ are dimensionless parameters. A describes the asymmetry between the daughter branches and is given by $A=-15.47\times [({\left(D_{1}^{h}\right)}^{2}-{\left(D_{2}^{h}\right)}^{2})/({\left(D_{1}^{h}\right)}^{2}+{\left(D_{2}^{h}\right)}^{2})]\times [(1-H_{d})/D_{P}^{h}]$. In this study, A=0 because the bifurcations are symmetric ($D_{1}^{h}=D_{2}^{h}$). B denotes the sigmoidal shape of the fitting function and is expressed by $B\;=\;1+8.13\times (1-H_{d})/D_{P}^{h}$. $X_{0}$ defines the minimal blood flow fraction $\Phi^{i}$, which is required to draw RBC into the daughter branch. It is computed as $X_{0}\;=\;1.12\times (1-H_{d})/D_{P}^{h}$. The numerical values for the parameters A, B, and $X_{0}$ were finally multiplied by a correction factor^47^ $MCV_{ratio}\;=\;\sqrt[{3}]{56/91}=0.85$ to account for the volume difference between porcine and human RBCs.

Next to the definitions for B and $X_{0}$ given by Pries *et al.*,^42^ we tried to find the best set of parameters ($\hat{B}$ and ${\hat{X}}_{0}$) by minimizing the Root Mean Square Error (RMSE) between the present *in vitro* results and the model of Pries *et al.*^42^ [Eq. (13)]. To this end, two parameters α and β were introduced as

$$\begin{array}{c} {\hat{X}}_{0}\;=\;\alpha \times X_{0}\;\mathrm{for}\;0<\alpha <2,\\ \hat{B}\;=\;\beta \times B\;\mathrm{for}\;0.6<\beta <2. \end{array}$$ (14)

To analyze the blood flow heterogeneity at individual bifurcations, the relative velocity difference between the daughter branches was computed according to Schmid *et al.*^49^ as

$$|\Delta^{r}U_{db}|\;=\;\frac{2\times |U_{rbc}^{1}-U_{rbc}^{2}|}{U_{rbc}^{1}+U_{rbc}^{2}},$$ (15)

where $U_{rbc}^{1}$ and $U_{rbc}^{2}$ are the velocities in the daughter vessels of a diverging bifurcation.

Likewise, we computed the average balance factor $\bar{B_{db}}$ for diverging bifurcations introduced by Schmid *et al.*^48^ according to

$$\bar{B_{db}}\;=\;\frac{1}{n}\sum_{k=1}^{n}\left(1-\frac{\left|0.5-\Phi_{k}\right|}{0.5}\right),$$ (16)

where $\Phi_{k}$ is the blood flow rate fraction for one of the daughter branches of the kth bifurcation and n is the total number of diverging bifurcations. The average balance factor $\bar{B_{db}}$ ranges from 0 for a completely unbalanced network to 1 for a perfectly balanced network. A network with $\bar{B_{db}}>0.8$ was considered well balanced.^48^

#### 3. RBC-free channels: Computation of plasma velocity by mass balance

**FIG. 4.**
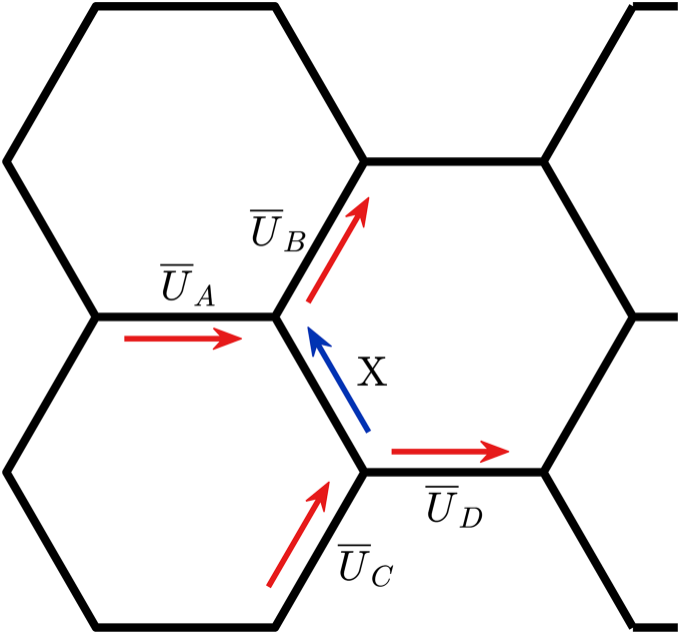
Computation of the plasma flow rate by mass balance. The magnitude and direction of plasma flow were evaluated by computing the mass balance for each of the two bifurcations sharing the RBC-free vessel.

There were four channels (Fig. 3, blue arrows) that carried no RBCs at all. Therefore, they required a different method for the computation of the flow rate. To this end, we estimated the flow velocity X in the RBC-free branch (Fig. 4) from mass balance equations at the adjacent bifurcations. To reduce the effect of measurement errors, the estimates from two neighboring bifurcations were averaged. This resulted in

$$X\;=\;\frac{\left({\bar{U}}_{B}-{\bar{U}}_{A})+({\bar{U}}_{C}-{\bar{U}}_{D}\right)}{2},$$ (17)

where ${\bar{U}}_{A},\;{\bar{U}}_{B},\;{\bar{U}}_{C},$ and ${\bar{U}}_{D}$ represent the mean RBC velocities according to Fig. 4 (data are reported in Appendix A). Note that the mass balance is formulated here with velocities because the cross sections are equal for all branches of the network.

## III. RESULTS

First, velocity measurements are validated against the Line Scan Method^27^ (LSM) and the mass balance is verified at the bifurcations (Sec. III A). Second, results on the hematocrit distribution in the microfluidic network (Sec. III B) are shown. Finally, phase separation data for 21 diverging bifurcations and an analysis for the minimization of the root mean square error between present results and literature models are presented (Sec. III C). Because the microchannel network is symmetric with respect to the x axis, the results are only reported and discussed for to the upper half of the microfluidic device.

### A. Validation of particle tracking velocimetry

#### 1. Validation of velocity with line scan method

**FIG. 5.**
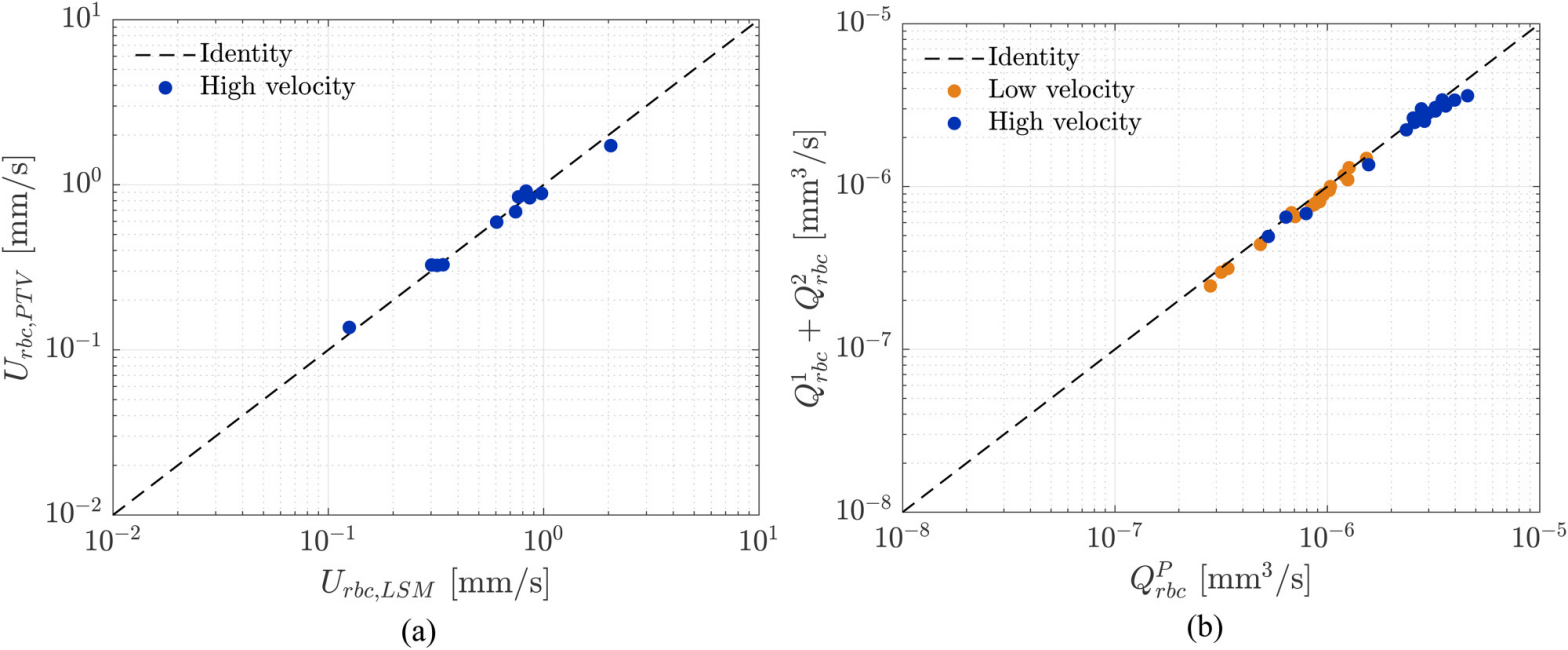
Method validation: (a) Velocity measurement. Comparison between Particle-Tracking Velocimetry (PTVlab) and Line Scan Method (LSM). Mean velocity data from a sample of 11 diverging bifurcations for the high velocity experiment were compared. (b) Mass balance. Verification of the RBC flux conservation for all diverging bifurcations considered in this study for low (orange) and high velocity experiment (blue). $Q_{rbc}^{P}$ is the RBC flux in the parent vessel, while $Q_{rbc}^{1}+Q_{rbc}^{2}$ is the sum of the RBC fluxes in the daughter branches.

The measured RBC velocities varied between 0.027 mm/s and 0.400 mm/s for the low velocity experiment. For the high velocity experiment they ranged between 0.051 mm/s and 1.726 mm/s. We validated these measurements against the Line Scan Method (LSM), which can be considered a reliable method in the limit of low hematocrit,^47^ because the line trace associated to each RBC is clearly discernible and the computation of the RBC velocity as the slope of this line is a straightforward process.^27^ Our PTVlab results were validated by considering a sample of 11 parent branches from the high velocity experiment ($\Delta P_{2}\;=\;47.1\;\mathrm{mbar}$) because it represented the worst case scenario in which the resolution of the PTVlab data was the potential limiting factor for the correct quantification of RBC velocity. The results presented in Fig. 5(a) show good agreement between the methods. The average of the relative difference between PTVlab and LSM is 7.4%±4.4 (mean±SD). This justified the choice of the particle-tracking method for the RBC velocity for both experiments ($\Delta P_{1}\;=\;35.3\;\mathrm{mbar}$ and $\Delta P_{2}\;=\;47.1\;\mathrm{mbar}$).

#### 2. Validation of RBC flux by mass balance

The method for the velocity computation was additionally tested by verifying that the presented data were consistent with the RBC flux mass balance at diverging bifurcations. For all diverging bifurcations with RBCs flowing in both daughter branches, the RBC flux in the parent branch was compared with the sum of the flux measured in the daughter branches. Results in Fig. 5(b) show good agreement between inflow and outflow at diverging bifurcations. The presented results indicate that the velocity measurements were accurate and respected mass conservation.

Following Roman *et al.*,^47^ an apparent deviation from mass conservation was computed for each diverging bifurcation as

$$E\;=\;\frac{\left|Q_{rbc}^{P}-(Q_{rbc}^{1}+Q_{rbc}^{2})\right|}{Q_{rbc}^{P}},$$ (18)

where $Q_{rbc}^{P}$ is the RBC flux in the parent branch and $Q_{rbc}^{1}$ and $Q_{rbc}^{2}$ are the RBC fluxes in the daughter branches. The mean deviation for the low velocity experiment is $\bar{E}\;=\;7.18\text{\%}\pm 3.52$ (mean±SD, range 1.65%–12.90%). The mean deviation for the high velocity experiment is $\bar{E}\;=\;7.51\text{\%}\pm 4.28$ (mean±SD, range 1.61%–14.64%). These results are in the range of other experimental studies^47,53^ with a similar approach for the RBC flux computation. The deviation from mass conservation inherent to the experimental nature of the study explained *a posteriori* the need of correction factors ($W_{P}$, $W_{1}$, and $W_{2}$, Sec. II D 2) introduced by Pries *et al.*^41^ for the correct quantification of phase separation at diverging bifurcations. The mean deviations computed in this study are in the same range as the results reported by Pries *et al.*^41^

### B. Network results: Hematocrit and RBC flux distribution

The distribution of RBCs and the flow field were analyzed for both experiments, which provided the basis for the investigation of the phase separation. The first step was the RBC tracking to identify preferential pathways within the network. Even if the number of RBCs in microchannels was time-dependent, the direction of flow was always constant and no inversion of flow direction was observed during the experiments. Figure 3 shows the flow directions for each branch of the network.

**FIG. 6.**
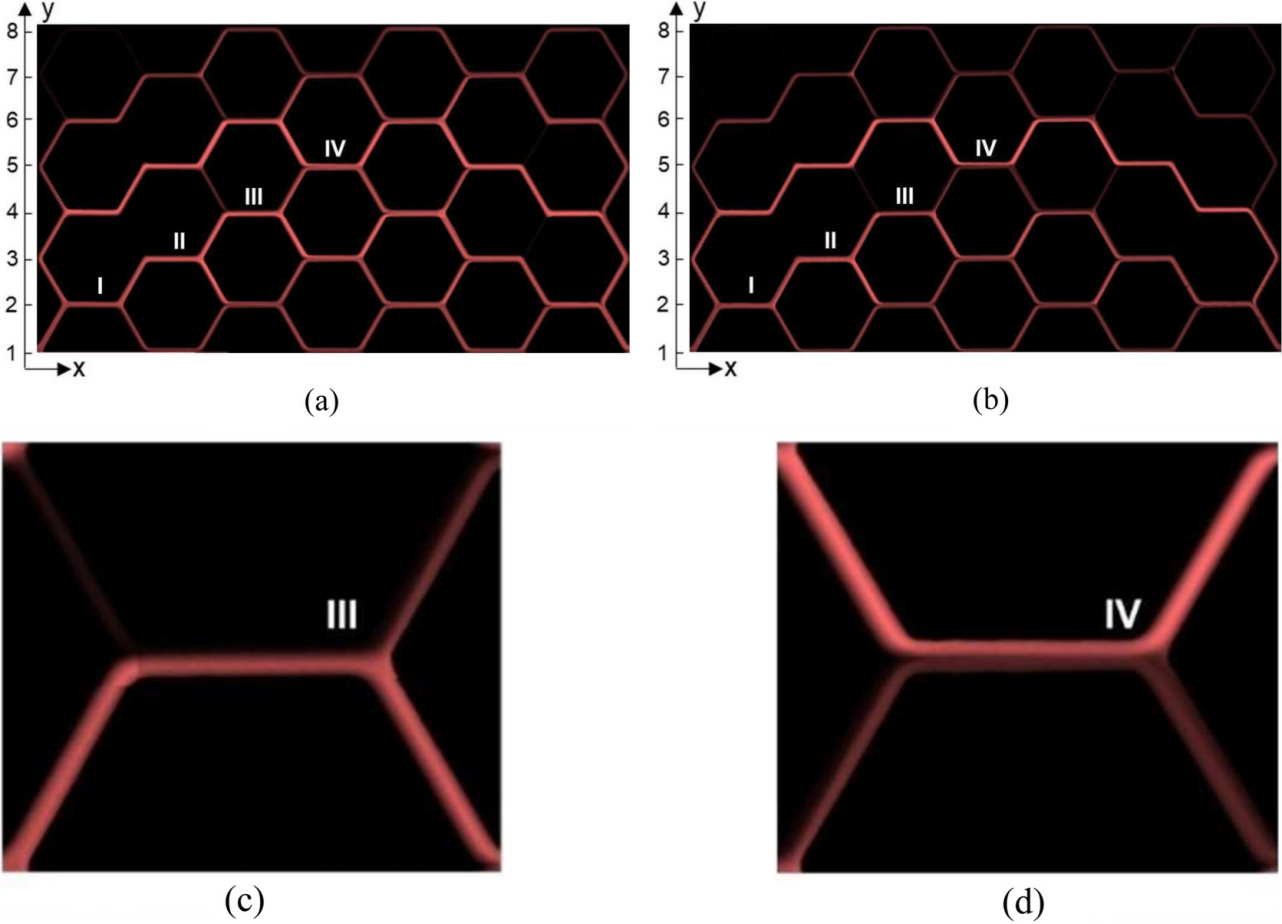
Hematocrit maps. Hematocrit distribution illustrated by the time-averaged pixel intensity of the video frames for the low velocity experiment (a) and for the high velocity experiment (b). The color intensity is monotonically related to the hematocrit of each branch: black denotes $H_{t}=0$, while red indicates $H_{t}=H_{t,\max}$. The pixel intensity was normalized with respect to the maximum intensity found for each experiment. Magnified images of bifurcation III (c) and IV (d) are displayed for the high velocity experiment.

The second step was the conversion of the time-averaged number of RBCs to the tube hematocrit (Sec. II D 1). The hematocrit distribution is heterogeneous in both experiments: there is an accumulation of RBCs in the center of the network, while there is a RBC reduction in the corners. Figure 6 shows the time-averaged normalized pixel intensity of the video frames for the low velocity experiment (a) and for the high velocity experiment (b), which provides a direct visual impression of the heterogeneous hematocrit distribution in the network with preferred RBC pathways and RBC-free branches.

**FIG. 7.**
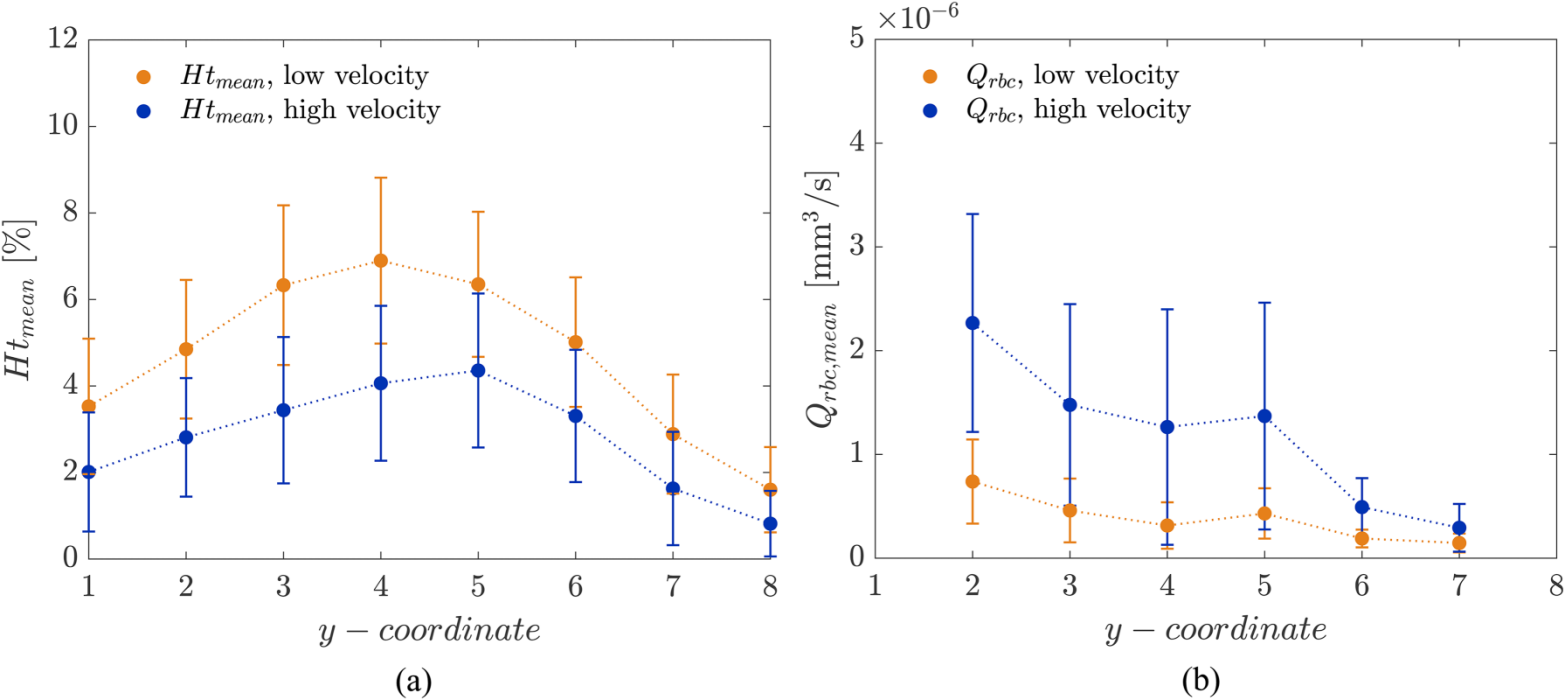
(a) Mean hematocrit. Time-averaged hematocrit in the function of the y-coordinate in the network (see Fig. 3) for either low (orange) and high velocity (blue) experiments. (b) RBC flux. Mean RCB flux in the parent vessels of all considered diverging bifurcations in function of the y-coordinate. Data are represented as mean (dots) and standard deviation (solid lines). Dotted lines are linear interpolations.

The hematocrit distributions expressed in function of the y-coordinate [Fig. 7(a)] are similar for both experiments. The distributions show that the main RBC accumulation did not occur close to the axis of symmetry, but in the central region (4 ≤ y ≤ 5) of the half-network.

Similar to the hematocrit distribution, the RBC flux [Fig. 7(b)] is also heterogeneous. Apart from the expected result that the mean RBC flux increases with the perfusion pressure and the flow velocities, the RBC flux is higher close to the axis of symmetry and decreases toward the periphery of the network. A RBC flux reduction of 80.2% and 87.1% was computed from the center (y=1) to the periphery of the network (y=8) for the low velocity and high velocity experiment, respectively.

### C. Phase separation

#### 1. Phase separation effect: Comparison with literature models

**FIG. 8.**
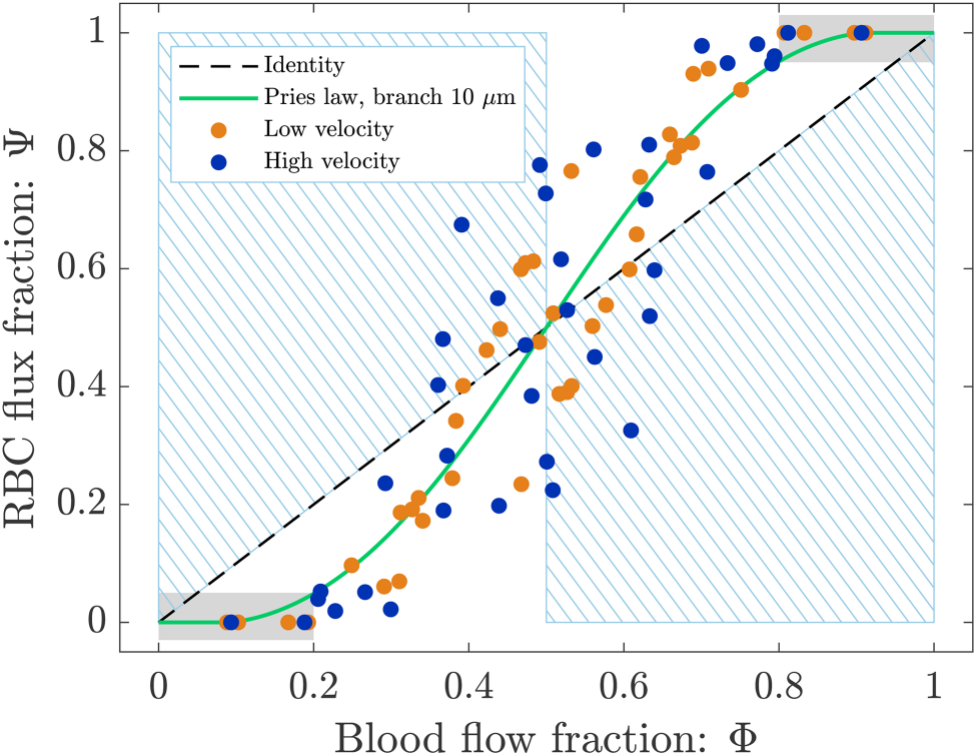
Phase separation results. Blood flow fraction vs RBC flux fraction data for low (orange) and high (blue) velocity experiments. Circles represent data from this study. The green continuous line is the empirical law by Pries *et al.*^42^ The dashed line is the identity line representing the proportional separation of blood and RBC (no phase separation). Data in the blue shaded regions indicate an inversion of the Zweifach-Fung effect.^10,52^ Data in the gray regions were sampled from bifurcations with one RBC-free daughter branch (see Figs. 3 and 4).

In order to study the phase separation, the RBC flux fraction $\Psi^{i}$ in each daughter branch of the diverging bifurcations was plotted in the function of the respective blood flow fraction $\Phi^{i}$ (Fig. 8). The results were compared with Pries’ separation law^41^ (empirical fitting of *in vivo* data with a sigmoid function).

Pries’ law appears to agree well for the experimental results obtained for both perfusion pressures. The majority of the data points follows the Zweifach-Fung effect and indicates that branches with lower blood flow fraction ($\Phi^{i}<0.5$) receive even less RBC flux ($\Psi^{i}<\Phi^{i}$) and vice versa. Exceptions to this general rule are represented by data in the blue shaded regions in Fig. 8. In this case, the bifurcation behavior is reversed: the branch with a lower blood flow fraction ($\Phi^{i}<0.5$) is receiving more RBCs ($\Psi^{i}>\Phi^{i}$) and vice versa. This phenomenon, known as inversion of the Zweifach-Fung effect^10,52^ or reverse partitioning,^5^ has been previously reported in experimental studies for single asymmetric bifurcations^10^ and simplified rectangular networks.^52^ To the best of our knowledge, it is reported here for the first time for a larger and more realistic *in vitro* network. The experimental data shown here are in good agreement with the numerical results reported by Balogh and Bagchi^5^ on RBC partitioning in simulated microvascular networks with *in vivo* like features.

Data points in the gray regions in Fig. 8 represent diverging bifurcations with one RBC-free daughter branch. Following the procedure explained in Sec. II D 3, the plasma flow rate was computed in the RBC-free branch (data for all variables of interest are reported in Appendix A). This result indicates that there exists a flow threshold below which no RBCs are entering a branch. This threshold corresponds to the parameter $X_{0}$ in Pries’ law. For both experiments, a flow threshold $X_{0}\;=\;0.19$ was observed, which means that for a blood flow fraction $\Phi^{i}<0.19$, we found a RBC flux fraction $\Psi^{i}=0$ and, correspondingly, $\Psi^{i}\;=\;1$ for $\Phi^{i}>0.81$.

The local phase separation at diverging bifurcations has consequences on the flow velocities at the global scale of the whole network. Table I shows that for the low velocity experiment, 41.2% of the bifurcations had a velocity difference $|\Delta^{r}U_{db}|<40\text{\%}$ while only 11.8% of the bifurcations had $|\Delta^{r}U_{db}|>80\text{\%}$ (Sec. II D 2). For the high velocity experiment, the blood flow distribution was more heterogeneous: 35.3% of bifurcations had a $|\Delta^{r}U_{db}|>80\text{\%}$. These results were supported by the averaged balance factor $\bar{B_{db}}$ [Eq. (16)] for the whole network. $\bar{B_{db}}\;=\;0.79$ and $\bar{B_{db}}\;=\;0.70$ were found for the low velocity experiment and for the high velocity experiment, respectively. These results indicate that the network is less balanced at higher velocities.

**TABLE I.** Relative velocity difference. Relative velocity difference between the daughter branches of each diverging bifurcation considered in the study. The relative velocity difference was grouped into three categories and the number of bifurcations for each category was counted.

Experiment	$0\text{\%}<|\Delta^{r}U_{db}|<40\text{\%}$	$40\text{\%}<|\Delta^{r}U_{db}|<80\text{\%}$	$|\Delta^{r}U_{db}|>80\text{\%}$
Low velocity	7	8	2
High velocity	6	5	6

#### 2. Influence of the RBC distribution in the cross sections of the parent vessels of a series of diverging bifurcations

**FIG. 9.**
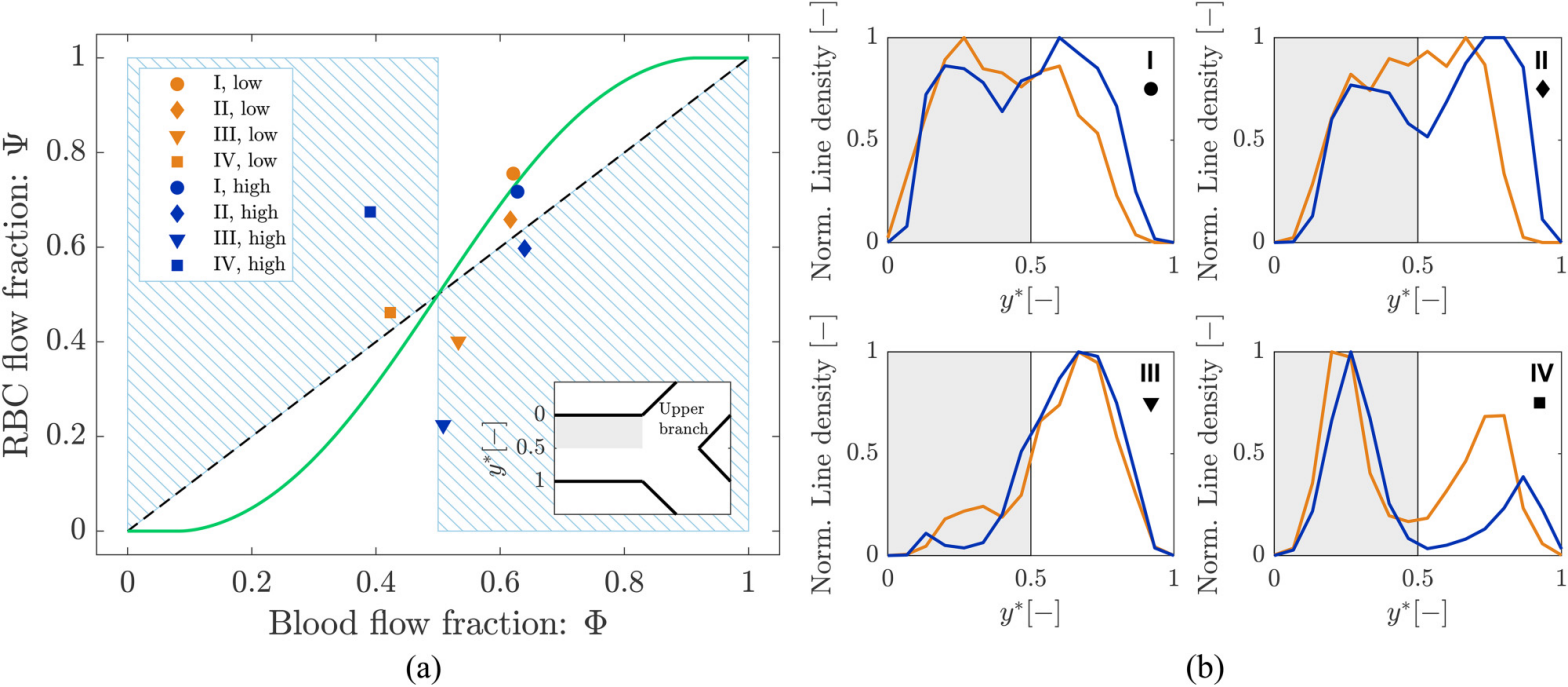
(a) Phase separation results. Blood flow fraction-RBC flux fraction data for the bifurcations I, II, III, and IV for low (orange) and high (blue) velocity experiments (extracted from Fig. 8). For all cases, data are sampled from the daughter branch closer to the upper half of the parent vessel [0<y∗<0.5, gray regions in the inset of panel (a) and in panel (b)]. The green continuous line is the empirical law from Pries *et al.*^42^ The dashed line is the identity line representing the proportional separation of blood and RBC (no phase separation). Data in the blue shaded regions indicate the inversion of the Zweifach-Fung effect.^10,52^ (b) Normalized RBC line density. Samples of normalized line density along the normalized width of the microchannels ($y^{*}=y/W_{ROI}$) for the parent vessels of the diverging bifurcations I, II, III, and IV (see Fig. 3). Continuous orange lines and blue lines represent low velocity and high velocity experiments, respectively.

The path along the bifurcations I, II, III, and IV indicated in Fig. 3 and in Fig. 6 features a series of diverging and converging bifurcations. Figure 9(a) presents data extracted from Fig. 8 corresponding to the diverging bifurcations I, II, III, and IV. For the shown results, the data were sampled from the daughter branch, which is closer to the upper half of the parent vessel [0<y∗<0.5, gray region in the inset of Fig. 9(a)].

In general, the phase separation behavior for bifurcations closer to the inlet of the microdevice (Bif. I and II) was reasonably well predicted by Pries’ separation law for both experiments. For the more distal bifurcations III and IV the deviation from Pries’ law increased and we observed an inversion of the Zweifach-Fung effect. This effect was stronger for the high velocity experiment.

A correlation was found between the phase separation results for the bifurcations I, II, III, and IV and the local hematocrit distribution in their respective parent vessels. Figure 9(b) shows that the normalized line density profiles underwent significant shape changes from the first to the last bifurcation (see also Appendix B). For the low velocity experiment, the profile in the parent vessel of bifurcations I was symmetric and almost flat while it was skewed with asymmetric peaks for bifurcations III and IV. Results from the high velocity experiment showed a similar shape transition with an even more skewed profile at the last bifurcation.

To quantitatively demonstrate the relation between the skewness and RBC partitioning, all divergent bifurcations were sorted based on the type of partitioning they presented (i.e., *classical* or *reversed*) and the mean hematocrit skewness index (${\bar{S}}_{h}$) was computed for each of the two groups. We found a ${\bar{S}}_{h,classical}\;=\;0.11\pm 0.1$ and ${\bar{S}}_{h,reverse}\;=\;0.21\pm 0.1$ for the bifurcations with classical and reverse partitioning, respectively. The Wilcoxon rank sum test was performed to compare the hematocrit skewness index for the two groups of bifurcations (significance for p <0.05). p=0.014 was obtained, meaning that the bifurcations with reverse partitioning have a statistically higher hematocrit skewness index.

In conclusion, for flat and symmetric line density profiles the RBC partitioning is more likely to follow the Zweifach-Fung effect and Pries’ separation law. For skewed line density profiles, the RBCs rather adhere to the streamlines and the phase separation is less sensitive to blood flow fraction differences.^6,51^ As a result, reverse partitioning is more likely.

#### 3. Analysis for the minimization of the root mean square error between present results and literature models

The phase separation model proposed by Pries *et al.*^41^ was based on the empirical fitting of *in vivo* data from arterial bifurcations in which the flow rate of the daughter branches was manually imposed by occluding a downstream vessel. In this study, the flow rates of daughter branches at diverging bifurcations was a natural consequence of the downstream microchannel resistances and the discrete contributions to resistance due to RBCs.

**FIG. 10.**
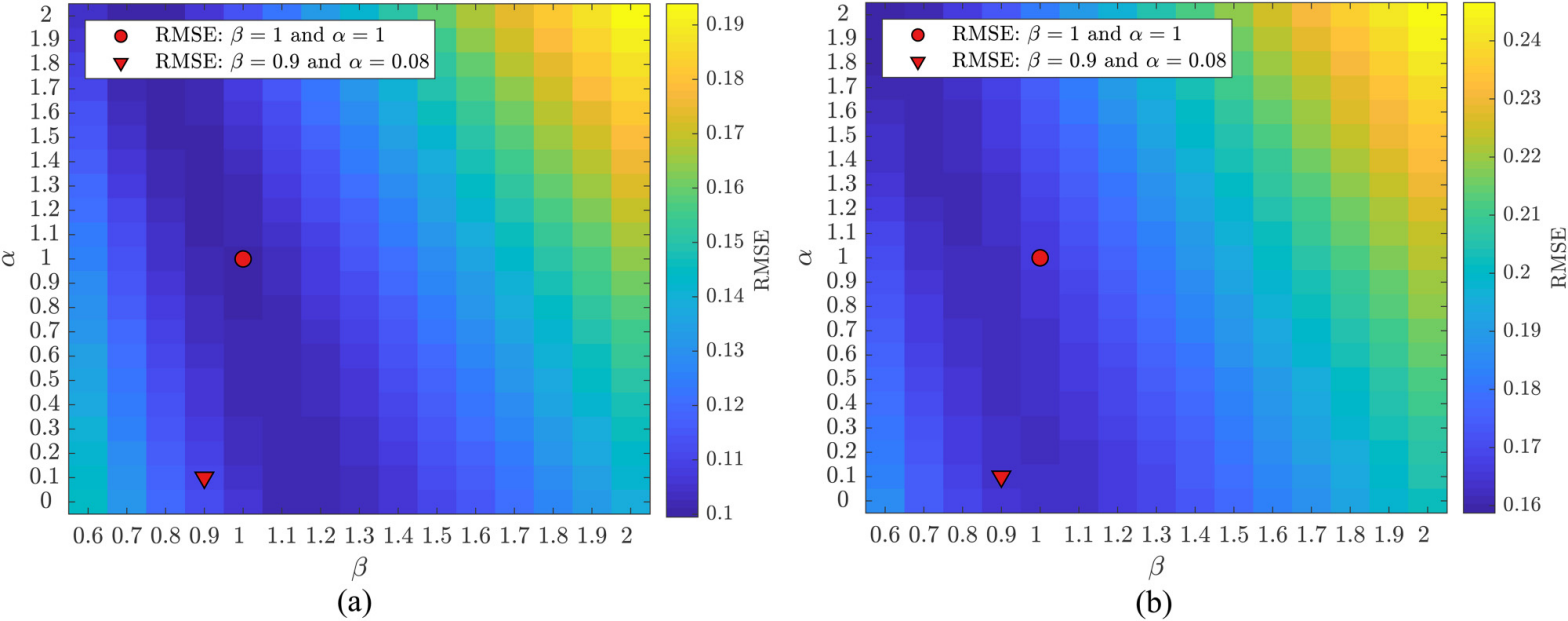
Root Mean Square Error (RMSE). RMSE between our data [low velocity (a) and high velocity data (b)] and the original phase separation law proposed by Pries *et al.*^42^ in the function of the parameters α and β. The filled circle represents the RMSE between the data present in this study and the original Pries’ phase separation law. The triangle represents the RMSE between the data presented in this study and Pries’ phase separation corrected according to the Bayesian *posthoc* analysis by Rasmussen *et al.*^43^

A quantification of the Root Mean Square Error (RMSE) was performed in order to understand to which extent the phase separation law of Pries^41^ is able to fit the present experimental data and to find the best set of parameters (${\hat{X}}_{0}$ and $\hat{B}$) to describe the *in vitro* data. According to the methodology presented in Sec. II D 2, colormaps were produced to quantify the RMSE in the function of two parameters α and β (Fig. 10). The RMSE for the original Pries’ curve (α=β=1) is $RMSE_{low}\;=\;0.101$ and $RMSE_{high}\;=\;0.166$ for the low and high velocity experiment, respectively.

The RMSE was also quantified for an updated version of the Pries’ separation law proposed by Rasmussen *et al.*^43^ By exploiting a Bayesian data analysis framework (*posthoc* analysis), a new set of parameters ($X_{0}$ and B) was proposed to better fit *in vivo* data with the well-known sigmoid function of Pries. Using the conventions of this study, the set of parameters introduced by Rasmussen *et al.*^43^ corresponds to ${\hat{X}}_{0}\;=\;\alpha \times X_{0}$ and $\hat{B}\;=\;\beta \times B$ with α=0.08 and β=0.9 resulting in $RMSE_{low}\;=\;0.110$ and $RMSE_{high}\;=\;0.171$.

## IV. DISCUSSION

In a recent review, the need for more experimental data to validate computational models of RBC flow in capillary networks^16^ was highlighted. To help fill this gap, the present study featured a microvascular network model with complex yet idealized topology with microchannels 10μm wide or less. The impact of RBCs on the global dynamics of the network as well as on the local hemodynamics at diverging bifurcations was investigated. To the best of our knowledge, *in vitro* quantitative phase separation data from such networks have not been reported yet.

On the scale of the whole network, the hematocrit (Fig. 6) and the RBC flux distribution [Fig. 7(b)] showed that the RBC flow was heterogeneously distributed throughout the network and preferential pathways were present. The basic mechanisms leading to preferential pathways are not specific to this network and they are a consequence of the effective hydraulic resistance of the microchannels and the phase separation law.^39^ It should be highlighted that these pathways developed freely in an *in vitro* microvascular network model and they were not a consequence of locally imposed boundary conditions on single bifurcations. The results on the relative velocity difference (Table I) and the average balance factor $\bar{B_{db}}$ of 0.79 (low velocity) and 0.70 (high velocity) (Sec. III C 1) suggest that the self-regulation process described in Sec. I was less effective for higher velocities. In their numerical study with a hexagonal network similar to ours, Schmid *et al.*^48^ reported that the average balance factor ranged between $0.7<\bar{B_{db}}<0.8$ for an inlet tube hematocrit $H_{t,inlet}<0.10$, which is comparable to our experimental data. The maximum in the balance factor ($\bar{B_{db}}\approx 0.84$) was obtained for $H_{t,inlet}\;=\;0.25$. As suggested by Schmid *et al.*,^48^ a sufficient number of RBCs in the network is needed to balance the downstream bifurcations. Considering that the maximum inlet tube hematocrit calculated in the present experimental study was $H_{t,inlet}<0.10$, it is reasonable to believe that the efficiency of self-regulation mechanisms was limited.

We found that the RBC splitting at diverging bifurcations is not proportional to the blood flow fraction in the daughter branches, which agrees with the consensus in the literature that phase separation at diverging bifurcations is dominated by the Zweifach-Fung effect.^5,41,47,52,53^ The data from the low velocity experiment can be well described by the empirical fitting of *in vivo* measurements proposed by Pries *et al.*,^41^ the RMSE is consistent with similar studies found in the literature.^47^ The data from the high velocity experiment are in reasonable agreement with Pries’ separation law for Φ<0.4 or Φ>0.6 (see Fig. 8). A deviation is present for 0.4<Φ<0.6 that might originate from the inversion of Zweifach-Fung effect^10,52^ and the *history effect*^34^ as explained further below. Results suggest that at most branching points the majority of RBCs is more likely to enter the high-flow branch (*classical partitioning*). In some bifurcations, RBCs preferred to enter the low-flow branch (blue shaded regions of Fig. 8). This phenomenon is a representation of the *reverse partitioning* (i.e., inversion of the Zweifach-Fung effect) and we found it to be more likely for higher flow velocities. This dependence on the flow velocity is consistent with the findings of an *in vitro* study with asymmetric single bifurcations.^10^

A complete description of phase separation is not trivial and in the limit of low hematocrit, two competing phenomena affect the RBC partitioning: either RBCs enter the branch with a higher flow rate or they follow the streamlines.^50^ It was demonstrated in a computational model^6^ that RBCs flowing along the centerline of the channel adhere to the Zweifach-Fung effect, while RBCs located closer to the walls more likely follow the streamlines of the plasma flow. In our experiments, both phenomena were likely to be present because the particle to channel diameter ratio was equal to 0.6, meaning cell margination or cell lateral focusing were possible.

A path featuring multiple diverging bifurcations was considered in Fig. 9 and the relation between phase separation and RBC spatial distribution in the parent vessel was investigated. We found that the RBCs are more likely to split according to the Zweifach-Fung effect if their distribution in the parent vessel is symmetric and homogeneous [Fig. 9(b), Bif. I]. By contrast, if the RBC distribution is skewed in the parent vessel [Fig. 9(b), Bif. III] or if the RBCs are marginated [Fig. 9(b), Bif. IV], it is more likely that they follow the streamlines and enter the closer branch. The latter situation supports the hypothesis^10^ that a depletion in the central region of the parent vessel may affect the phase separation by reducing the number of RBCs entering the high-flow branch (i.e., reducing the Zweifach-Fung effect). As an example, the upper daughter branch of bifurcation IV [blue rectangle in Fig. 9(a)] received a higher RBC flux fraction than the blood flow fraction because in the parent vessel, the RBC line density was much higher on the upper side of the parent vessel [0<y∗<0.5, Fig. 9(b)]. The peculiar shape of the line density profiles for bifurcations III and IV has to be attributed to the fact that the RBC distributions in these parent vessels are in turn the result of two separate RBC distributions from converging branches. The skewed profile in the parent of bifurcation III is the result of the confluence of a channel with very low hematocrit and a channel with very high hematocrit [Fig. 6(c)]. The bimodal distribution in the parent of bifurcation IV is probably due to a lack of mixing of the converging RBC fluxes at the inflow to this vessel [Fig. 6(d)]. This interpretation is consistent with numerical and experimental results reported for a Y-shaped converging bifurcation, where a cell-depleted layer of RBC was found in the center of a microchannel after a confluence.^3,29,34^ Moreover, we observed a statistically significant higher hematocrit skewness index for the reverse partitioning and a greater deviation from Pries’ law (i.e., *reverse partitioning*) for the high velocity. Thus, the hematocrit skewness is higher in the high velocity experiment. The local mechanisms that may explain the origin of the skewness and its velocity dependence are probably related to the dynamics of RBCs, which is governed by the complex interaction between lift force, shear rate gradient experienced by the cell and, in the case of concentrated RBC suspension, nonlinear anisotropic shear-induced diffusion.^19,32,37^ It is the resultant of these mechanisms at RBC scale that may lead to skewed hematocrit profiles.

*In vitro* studies of phase separation are mostly performed in single bifurcations in which the velocity and hematocrit profiles in the parent vessel are fully developed and symmetric.^12,14,26,47,53^ Unfortunately, this configuration may not necessarily be a good representation of the hemodynamics *in vivo*. In the case of a complex network with diverging/converging bifurcations, the hematocrit profile disturbed by the flow field at a first bifurcation may not be able to regain symmetry before the next bifurcation.^9,32,53^ The length required to regain complete symmetry was estimated to be even greater than 11 diameters for disturbed flow conditions.^38^ The low radial RBC dispersivity^53^ and the short time a RBC spends within the channel provide evidence that the flow history can be important if the bifurcations are close to each other or if the flow rates are sufficiently high.^9^ The possibility to compute RBC line density profiles along a path with multiple diverging bifurcations allowed us to study this phenomenon and it indicated that a *history effect*, as described by Merlo,^34^ is indeed present in our model of a microvascular network. A skewed RBC distribution across the parent vessel does not have enough time to regain symmetry and leads to reversed cell partitioning, which is unlikely to be seen in a model of single bifurcation with homogeneous RBC distribution in the parent vessel. A detailed analysis can be found in Appendix C to demonstrate that an equilibration of the hematocrit profile between two successive bifurcations was unlikely to be achieved in the present microchannel network.

The agreement of the present *in vitro* results on the phase separation with the computational results of Balogh and Bagchi^4,5^ is striking. Both studies highlighted the simultaneous occurrence of classical and reverse partitioning in a microvascular network and related these phenomena to the upstream hematocrit distribution and to the influence of sequential bifurcations. Our experimental approach allowed to overcome a limitation of their numerical study, namely, the modeling of the RBC membrane. In their model, Balogh and Bagchi did not consider the membrane viscosity, which has an impact on the time scale of shape recovery when the RBCs are deformed. This may have a negative consequence on the study of cellular mechanisms at the bifurcations. However, the consistency between experiments and simulations resolved this potential issue and validates the numerical model of Balogh and Bagchi.^4,5^

## V. LIMITATIONS

*In vitro* experiments involve simplifications with respect to *in vivo* investigations. Capillaries are cylindrical and nonuniform over their length while in our study we had straight microfluidic channels with rectangular cross sections. Our aim was to produce microchannels with a characteristic width ≤10μm to mimic the anatomical dimensions of the capillaries. Due to technological limitations, fabricating rectangular channels was the only available option (networks of microchannels with circular cross section are limited today to channel size 30−40μm).^10,30^ We are aware that rectangular cross sections may alter the local fluid dynamics: RBCs do not occupy the whole lumen of the channels leaving open regions at the corners for the plasma to flow. We tried to limit these effects by reproducing the confinement that RBCs experiences in the capillaries using channel sizes slightly bigger than the RBCs. Further, the surface properties of the internal walls of capillaries were not reproduced. *In vivo*, there is an endothelial surface layer with glycocalyx while *in vitro*, there is only a nonspecific adsorption of bovine serum albumin.^47^ The good agreement between our *in vitro* results and the empirical fitting of *in vivo* data indicated that these simplifications are nondominant determinants of phase separation as long as averaged variables are considered and cell-cell interactions are weak.^47^

Another limitation is related to the blood mimicking fluid used in the experiments. In the RBC solution, there were no other biological components than RBCs. This prevented the study of the interactions between blood components that may affect the cell screening and phase separation at bifurcations *in vivo*.

As reported by Roman *et al.*,^47^ the quite large experimental variability has to be accounted as another limitation. The experimental variability might be attributed to technical difficulties experienced with such small microchannels. For the same reason, we chose to report experiments with only a reservoir hematocrit $H_{d}\;=\;10\text{\%}$ because it was challenging (if not impossible) to perfuse the network in a controlled manner with more concentrated RBC suspensions. In fact, microchannel obstructions were more likely to happen at high hematocrit determining a perturbation in the network and biasing the study of the phase separation.

## VI. CONCLUSIONS

In the present study, we developed an *in vitro* microvascular network model to study the RBC phase separation at diverging bifurcations. For the first time, we provided quantitative data on the RBC distribution and separation in a complex *in vitro* network model. These data confirmed computational results on RBC partitioning in realistic microvascular networks.^5^

The data reproduced the classical result that branches receiving the higher blood flow fractions typically received an overproportional RBC flux fraction. An inversion of this classical phase separation behavior was observed in the case of a skewed hematocrit profile in the parent vessel of the bifurcations, especially for high-flow rates. This provided evidence that the flow history has to be considered to properly describe the RBC phase separation in complex networks with multiple diverging bifurcations. Finally, we were able to identify a critical blood flow fraction of Φ=0.19 below which only plasma enters a downstream branch.

In conclusion, the nonuniform RBC distribution and partitioning underline the importance of treating blood as a biphasic fluid and the need of considering the discrete and particulate nature of blood to properly describe fluid dynamics at the microscale, especially in microvascular networks where the RBC dynamics becomes complex and often counterintuitive.^39^

## Figures and Tables

**FIG. 11.**
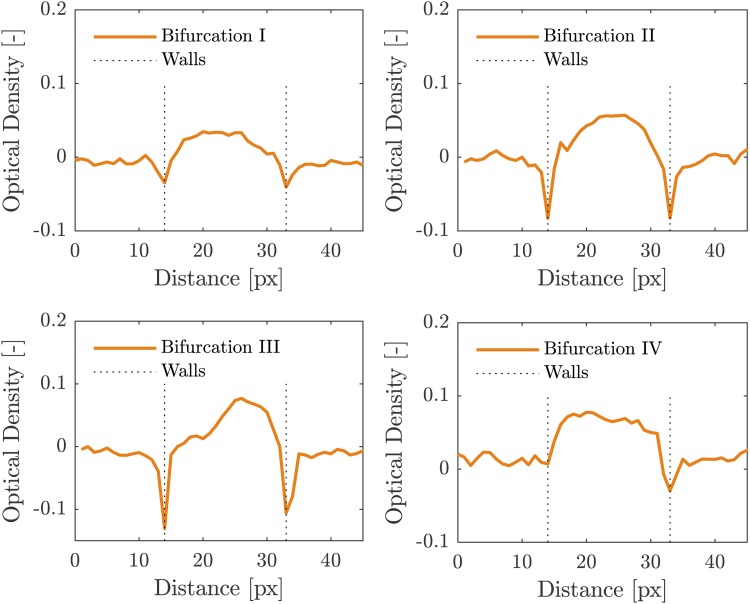
Optical density profiles. Optical density profiles for the low velocity experiment computed for the parent vessels of the diverging bifurcations I, II, III, and IV following Roman *et al.*^47^

**FIG. 12.**
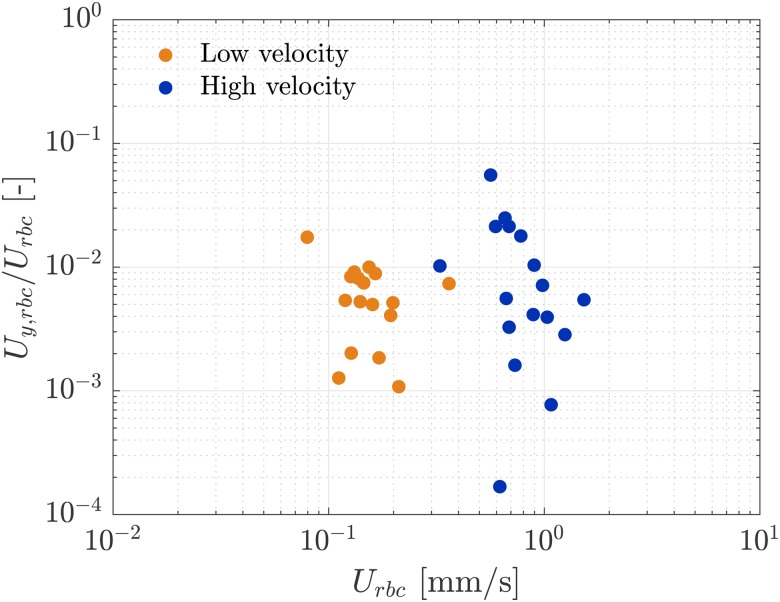
RBC velocity ratio. Ratio between the RBC velocity in the lateral direction ($U_{y,rbc}$) and the magnitude of the RBC velocity ($U_{rbc}$) for the low (orange) and high (blue) velocity experiment.

## APPENDIX A: RBC-FREE BRANCHES

Considering a pair of bifurcations sharing a RBC-free channel, the plasma flow was computed by mass conservation as

$$X=\frac{\left({\bar{U}}_{B}-{\bar{U}}_{A})+({\bar{U}}_{C}-{\bar{U}}_{D}\right)}{2},$$ (A1)

where ${\bar{U}}_{A},\;{\bar{U}}_{B},\;{\bar{U}}_{C},$ and ${\bar{U}}_{D}$ represent the mean RBC velocities according to Fig. 4. The raw data from the particle-tracking velocimetry that have been utilized to compute the mass conservation are reported in Tables II and III for the low and high velocity experiments, respectively. These data have been ultimately used to compute the blood flow fraction and the RBC flux fraction in the resulting diverging bifurcations with a RBC-free daughter branch. During the postprocessing of the image sequences, we noted that a spurious white blood cell flowed through the bifurcation named “*Q4, Bif. III*” during the high velocity experiment. For this reason, the velocity data were biased and this bifurcation was excluded from further analysis.

**TABLE II.** Time-averaged RBC velocity for the bifurcations with a RBC-free channel. Bifurcations sharing a RBC-free branch are pairwise grouped. These data refer to the low velocity experiment.

Bifurcation	RBC velocity (mm/s)			
Q1, Bif. IV, *low velocity*	${\bar{U}}_{A}=0.0793$	${\bar{U}}_{B}=0.0964$	${\bar{U}}_{B}-{\bar{U}}_{A}=0.0171$	*X* = 0.0158
Q1, Bif. V, *low velocity*	${\bar{U}}_{C}=0.1544$	${\bar{U}}_{D}=0.1398$	${\bar{U}}_{C}-{\bar{U}}_{D}=0.0146$
Q4, Bif. II, *low velocity*	${\bar{U}}_{A}=0.1375$	${\bar{U}}_{B}=0.1542$	${\bar{U}}_{B}-{\bar{U}}_{A}=0.0167$	*X* = 0.0138
Q4, Bif. III, *low velocity*	${\bar{U}}_{C}=0.0822$	${\bar{U}}_{D}=0.0714$	${\bar{U}}_{C}-{\bar{U}}_{D}=0.0108$
Q5, Bif. IV, *low velocity*	${\bar{U}}_{A}=0.1273$	${\bar{U}}_{B}=0.1502$	${\bar{U}}_{B}-{\bar{U}}_{A}=0.0229$	*X* = 0.0202
Q5, Bif. V, *low velocity*	${\bar{U}}_{C}=0.2282$	${\bar{U}}_{D}=0.2108$	${\bar{U}}_{C}-{\bar{U}}_{D}=0.0174$
Q8, Bif. I, *low velocity*	${\bar{U}}_{A}=0.1979$	${\bar{U}}_{B}=0.2339$	${\bar{U}}_{B}-{\bar{U}}_{A}=0.0360$	*X* = 0.0289
Q8, Bif. II, *low velocity*	${\bar{U}}_{C}=0.1529$	${\bar{U}}_{D}=0.1310$	${\bar{U}}_{C}-{\bar{U}}_{D}=0.0219$

**TABLE III.** Time-averaged RBC velocity for the bifurcations with a RBC-free channel. Bifurcations sharing a RBC-free branch are pairwise grouped. These data refer to the high velocity experiment.

Bifurcation	RBC velocity (mm/s)			
Q1, Bif. IV, *high velocity*	${\bar{U}}_{A}=0.3277$	${\bar{U}}_{B}=0.4098$	${\bar{U}}_{B}-{\bar{U}}_{A}=0.0821$	*X* = 0.0661
Q1, Bif. V, *high velocity*	${\bar{U}}_{C}=0.7054$	${\bar{U}}_{D}=0.6554$	${\bar{U}}_{C}-{\bar{U}}_{D}=0.0500$
Q4, Bif. II, *high velocity*	${\bar{U}}_{A}=0.8340$	${\bar{U}}_{B}=0.8474$	${\bar{U}}_{B}-{\bar{U}}_{A}=0.0107$	…
Q4, Bif. III, *high velocity*	${\bar{U}}_{C}=0.2949$	${\bar{U}}_{D}=0.3163$	${\bar{U}}_{C}-{\bar{U}}_{D}=-0.0231$
Q5, Bif. IV, *high velocity*	${\bar{U}}_{A}=0.6670$	${\bar{U}}_{B}=0.8409$	${\bar{U}}_{B}-{\bar{U}}_{A}=0.1739$	*X* = 0.1118
Q5, Bif. V, *high velocity*	${\bar{U}}_{C}=1.2571$	${\bar{U}}_{D}=1.2075$	${\bar{U}}_{C}-{\bar{U}}_{D}=0.0496$
Q8, Bif. I, *high velocity*	${\bar{U}}_{A}=1.1019$	${\bar{U}}_{B}=1.3526$	${\bar{U}}_{B}-{\bar{U}}_{A}=0.2507$	*X* = 0.1643
Q8, Bif. II, *high velocity*	${\bar{U}}_{C}=0.8729$	${\bar{U}}_{D}=0.7950$	${\bar{U}}_{C}-{\bar{U}}_{D}=0.0779$

## APPENDIX B: OPTICAL DENSITY PROFILES

A photometric method has been reported by Roman *et al.*^47^ to compute the tube hematocrit for high concentrations of RBCs. The same method has been applied here to qualitatively validate the line density profiles presented in Sec. III C 2. For each frame of the acquisition, the pixel intensity along the lateral direction Y at a fixed longitudinal position $x_{ref}$ was computed [$I(x_{ref},y)$]. The mean pixel intensity was obtained as the time-averaged intensity over all the frames of the acquisition. As a reference, the pixel intensity at the same longitudinal position was computed with only plasma in the channel [$I^{0}(x_{ref},y)$]. The light attenuation at any point ($x_{ref},y$) was defined as the ratio of $I^{0}(x_{ref},y)/I(x_{ref},y)$. The lateral optical density profile ($OD(x_{ref})$) was obtained by averaging along the lateral direction Y as follows:

$$OD(x_{ref})=\log{\left\langle \frac{I^{0}(x_{ref})}{I(x_{ref})}\right\rangle }_{y},$$ (B1)

where ${\left\langle \right\rangle }_{y}$ denotes the averaging along the transversal direction. Figure 11 shows the optical density profiles obtained for the bifurcations I, II, III, and IV for the low velocity experiment.

The hematocrit is linearly proportional to the optical density; therefore, the higher the *OD*, the higher the tube hematocrit at a specific lateral position. The optical density profiles are in reasonable qualitative agreement with the line density profiles presented in Fig. 9(b). These results demonstrate the validity of the method developed to obtain the line density profiles and the equivalence to another method found in the literature for the hematocrit computation.^34,47^

## APPENDIX C: ANALYSIS TO STUDY THE RBC EQUILIBRATION

A detailed analysis is performed to demonstrate that the “*history effect*” was present in the capillary network and the relaxation of the hematocrit profile between two successive bifurcations was not likely to be achieved due to the short length of the microchannels.

First, velocimetry data obtained with the particle tracking can be useful to quantitatively assess the contribution of the lateral RBC velocity (i.e., the velocity component in the y-direction) to the magnitude of the RBC velocity. Figure 12 shows the ratio between the lateral RBC velocity and the magnitude of the RBC velocity $U_{y,rbc}/U_{rbc}$ for all diverging bifurcations considered in our study as a function of the magnitude of the RBC velocity. The median ratio is $5.4\times 10^{-3}$ and $5.6\times 10^{-3}$ for the low and high velocity experiments, respectively. Given that the RBC velocity in the $U_{y,rbc}$ is on average three orders of magnitude smaller than $U_{rbc}$, it is reasonable affirm that the displacement in the lateral direction is very limited.

Second, a dimensional analysis can provide insight on the contribution of the RBC advection in the main flow direction with respect to the RBC dispersion in the lateral direction. Considering the length L of a microchannel and the mean RBC velocity $U_{rbc,mean}$ determined by particle-tracking, the advection time scale (i.e., the time for a RBC to pass through a channel of length L) is given by

$$T_{adv}=\frac{L}{U_{rbc,mean}}=\frac{85\times 10^{-3}\;\mathrm{mm}}{0.521\;\mathrm{mm}/s}=0.163\;s.$$ (C1)

Using the value of radial dispersivity reported by Cokelet^11^ ($D=10^{-7}\;{\mathrm{mm}}^{2}/s$), it is possible to compute the dispersion time scale (i.e., the time required by RBCs to disperse over the width W of the microchannel) as:

$$T_{disp}=\frac{W^{2}}{D}=\frac{10^{-4}\;{\mathrm{mm}}^{2}}{10^{-7}\;{\mathrm{mm}}^{2}/s}=10^{3}\;s.$$ (C2)

The fact that the related Peclet number $Pe=T_{adv}/T_{disp}\ll 1$ (which holds even if we had considered the minimum RBC velocity) indicates that the RBC flow is dominated by advection and the radial dispersion is of minor relevance in this configuration.

Third, by looking at single-cell migration models,^32^ we estimated the typical time a RBC requires to migrate from the wall (y∗=W/2) through the Cell-Free Layer (CFL) (y∗=(W/2)−d) as

$$\tau_{L}=\frac{(W/2)\times d^{\delta_{S}+1}}{U_{rbc,\max}\times \xi_{S}\times R_{0}^{\delta_{S}+1}}.$$ (C3)

Considering the optimized parameters provided by Losserand *et al.*^32^ and the geometrical dimensions of our microchannels ($\delta_{S}=1.3$, $\xi_{S}=1.1\times 10^{-2}$, $R_{0}=2.37\;\mu m$ for porcine RBCs, d=2μm, $U_{rbc,\max}=1.726\;\mathrm{mm}/s$, W/2=5μm), we obtained $\tau_{L}=0.178\;s$.

Similarly, we estimated the equilibration time due to the shear-induced diffusion as

$$\tau_{D}=\frac{{\left(W/2\right)}^{3}}{f\times U_{rbc,\max}\times H_{t}\times R_{0}^{2}}.$$ (C4)

Considering f=0.06 from Grandchamp *et al.*^19^ and $H_{d}=10\text{\%}$, we obtained $\tau_{D}=2.149\;s$.

Following Losserand *et al.*,^32^ the length a RBC requires to pass through the CFL is $\tau_{L}\times U_{rbc,\max}\approx 307.2\;\mu m$ and the one to reach equilibration is $\tau_{D}\times U_{rbc,\max}\approx 3.72\;\mathrm{mm}$, which is longer than the length of our microchannels (L=85μm). This is consistent with the results provided by Losserand *et al.*^32^ where lengths of 292.5μm and 930μm were needed to study the RBC transversal migration in microchannels 3.9μm and 6.2μm wide, respectively. Therefore, the hematocrit distribution relaxation is not likely to happen over the short distance between two successive bifurcations and an *history effect* is present in the microchannel network.
